# The Impact of GLP1 Agonists on Bone Metabolism: A Systematic Review

**DOI:** 10.3390/medicina58020224

**Published:** 2022-02-02

**Authors:** Ioanna Daniilopoulou, Eugenia Vlachou, George I. Lambrou, Anastasia Ntikoudi, Eleni Dokoutsidou, Georgia Fasoi, Ourania Govina, Anna Kavga, Athanasios N. Tsartsalis

**Affiliations:** 1 Department of Nursing, School of Health Sciences, University of West Attica, Ag. Spydironos 28, 12243 Athens, Greece; ioannadaniil88@hotmail.com (I.D.); nastaziantikoudi@gmail.gr (A.N.); edokout@uniwa.gr (E.D.); gfasoi@uniwa.gr (G.F.); ugovina@uniwa.gr (O.G.); akavga@uniwa.gr (A.K.); 2 Choremeio Research Laboratory, First Department of Pediatrics, National and Kapodistrian University of Athens, Thivon & Levadeias 8, 11527 Athens, Greece; glamprou@med.uoa.gr; 3 Department of Endocrinology Diabetes and Metabolism, Naval Hospital of Athens, Dinokratous 70, 11521 Athens, Greece

**Keywords:** GLP, GLP1RAs, bone mineral density, diabetes mellitus

## Abstract

*Background and Objectives:* The association between diabetes mellitus and increased risk of bone fractures has led to the investigation of the impact of antidiabetic drugs on bone metabolism. Glucagon-like peptide-1 receptor agonists (GLP1RAs) are a relatively novel and promising class of anti-hyperglycemic drugs. In addition to their blood glucose lowering action, GLP1RAs seem to have additional pleiotropic properties such as a beneficial skeletal effect; although the underlying mechanisms are not completely understood. The present systematic review summarizes current evidence about GLP1RAs and their effects on bone metabolism and fracture. *Methods:* An extensive literature search was conducted based on electronic databases namely, PubMed, Google Scholar and Cochrane Central Register of Controlled Trials (CENTRAL) through October 2019 to January 2020 for articles related to bone mineral density, diabetes mellitus and GLP1RAs. We included articles published in English. Finally, we included four randomized controlled trials, three meta-analyses, a case-control study and a population-based cohort analysis. *Results:* Based on the articles included, the animal studies indicated the salutary skeletal effects of GLP1RAs in opposition to what has been commonly observed in human studies, showing that these agents have no impact on bone mineral density (BMD) and the turnover markers. Moreover, it was demonstrated that GLP1 was not associated with fracture risk as compared to other anti-hyperglycemic drugs. *Conclusions:* Findings from this systematic review have demonstrated the neutral impact of GLP1RAs on BMD. Moreover, further double-blind randomized controlled trials are needed to draw more meaningful and significant conclusions on the efficacy of GLP1RAs on BMD.

## 1. Introduction

### 1.1. Glucagon-Like Peptide 1 (GLP1) and Bone Metabolism

**Figure 1:**
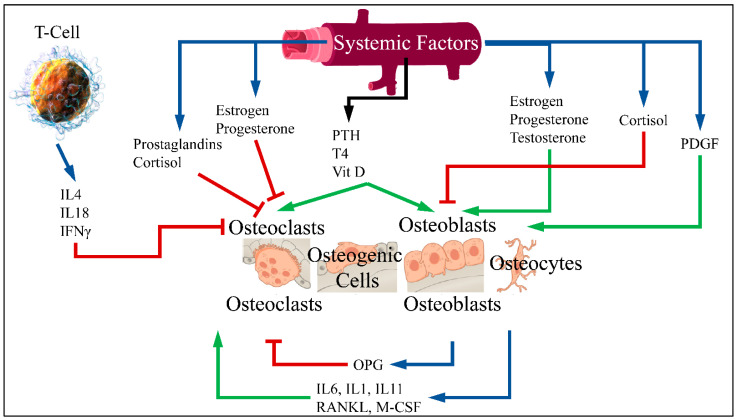
RANKL is an osteoclast differentiation factor. This factor binds to the RANK receptor in primary osteoclast cells and leads to differentiation and activation of osteoclasts. RANK enhances the action of osteoclast while OPG binds to RANKL and therefore inhibits bone resorption. An imbalance of OPG/RANKL/RANK expression is responsible for osteoporosis [6] (Legend: IL: Interleukins, IFNγ: Interferon gamma, PTH: Parathormone, T4: Thyroxine, Vit D: Vitamin D, PDGF: Platelet-Derived Growth Factor, OPG: Osteoprotegerin, RANKL: Receptor activator of nuclear factor kappa-Β ligand, M-CSF: Macrophage colony stimulating factor. Blue arrows imply production; red arrows imply inhibition; green arrows imply stimulation) (T-Cell is reproduced under CC BY 3.0 License from https://en.wikipedia.org/wiki/T_cell#/media/File:Blausen_0625_Lymphocyte_T_cell_(crop).png. Bone cells are reproduced under the CC BY 3.0 License from https://en.wikipedia.org/wiki/Bone#/media/File:604_Bone_cells.jpg. Vein is reproduced under the CC BY-SA 3.0 License from https://en.wikipedia.org/wiki/Vein#/media/File:Vein_(retouched).svg. All images last accessed on 26 September 2020).

Patients with diabetes mellitus are at an increased risk of fragility fractures [1]. Although many experimental studies have reported an association between diabetes and the risk of fracture, the connection between diabetes and osteoporosis has not been clarified [2]. Normal bones are constantly renewed through the process of breaking down old bone and generating new. The balance between breaking down and reformation is important for healthy bones [3]. The process of bone formation and bone resorption is called bone metabolism, in which the receptor activator of nuclear factor-κB ligand (RANKL)/receptor activator of nuclear factor-κB (RANK)/osteoprotegerin (OPG) pathway is the most crucial. Bone formation is performed by stimulating osteoblasts and inhibiting osteoclasts. Bone resorption is performed by an active osteoclast and stimulated by RANKL. RANKL is a cytokine belonging to the Tumor Necrosis Factor (TNF) family. This factor connects to RANK receptor in osteoclast progenitor cells and leads to differentiation and activation of osteoclasts. Moreover, RANKL is bound by OPG, secreted by osteoblasts, which inhibits osteoclast differentiation [4,5] (Figure 1).

An additional pathophysiological mechanism of GLP1 action, concerns the upregulation of RUNX2, alkaline phosphatase (ALP), collagen type-1 (COL1) and osteocalcin (OC), which are responsible for osteoblast stimulation [7]. This has been shown in a previous study on ovariectomized rats, where GLP1 agonist administration led to the upregulation of the aforementioned factors [7,8]. All previous studies agree on the fact that GLP1 can upregulate RUNX2, ALP, COL1 and OC as well as *N*-terminal propeptide of type I procollagen (P1NP) [9]. An additional mechanism through which GLP1 promotes bone formation is by regulating glucose metabolism. In particular, it has been found that hyperglycemia and lumbar BMD are reversely related, i.e., hyperglycemia is a factor reducing BMD. Thus, it has been speculated that GLP1 can indirectly affect bone metabolism by regulating glucose levels [10]. Another study has reported that GLP1 agonists, when binding to the GLP1 receptor (GLP1R), hydrolyze glycosylphosphatidylinositols, subsequently generating inositolphosphoglycans and therefore activating five important down-stream pathways, i.e., phosphatidylinositol-3 kinase (PI3K) and mitogen-activated protein kinase (MAPK) [11], as well as ERK1/2, p38 and JNK pathways [12].

**Figure 2:**
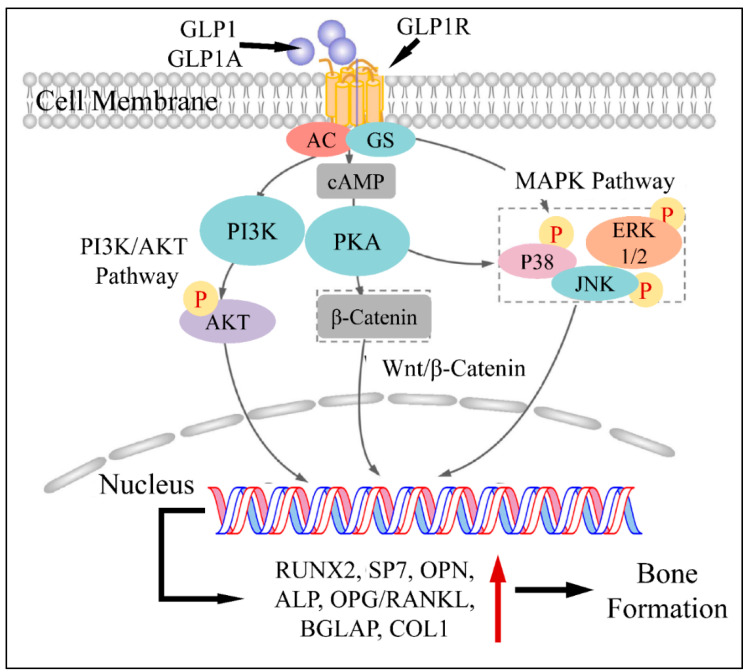
The mechanism of GLP1 and GLP1A in bone formation: GLP1 or GLP1A bind to GLP1R. The complex activates PI3K and PKA, which subsequently phosphorylates (*P*) AKT, PKA as well as P38, ERK1/2 and JNK. AKT enters the nucleus and regulates gene transcription. In addition, PKA induces β-catenin, which also enters the nucleus through the Wnt/β-catenin pathway. Similarly, phosphorylated P38, JNK and ERK1/2 enter the nucleus. All transcription factors facilitate gene expression, which leads to the induction of bone formation (Legend: GLP1: Glucagon-like peptide 1, GLP1A: Glucagon-like peptide 1 agonist, GLP1R: Glucagon-like peptide 1 receptor, GS: G-Proteins, cAMP: cyclic AMP, PI3K: Phosphoinositide 3-kinases, PKA: Protein kinase A, P38: p38 mitogen-activated protein kinase, ERK1/2: extracellular signal-regulated kinases, JNK: c-Jun *N*-terminal kinase, RUNX2: Runt-related transcription factor 2, SP7: Transcription factor Sp7, OPN: Osteopontin, ALP: Alkaline phosphatase, OPG: Osteoprotegerin, BGLAP: Osteocalcin gene, COL1: Type-1 collagen. Reproduced from Xie et al. (2021) [9] under the Creative Common License CC BY 3.0 link: https://www.frontiersin.org/articles/10.3389/fphar.2021.697442/full, accessed on 28 January 2022).

Another important role of GLP1 has been found to be related to the Wnt pathway. The canonical Wnt pathway, includes LPR5/6, β-catenin, GSK-3β and T cell factor activation which results in osteoblast differentiation and maturation [13,14]. GLP1 has been found to be a direct stimulator of these pathways and factors and therefore a significant regulator of bone metabolism. Sclerostin, encoded by the *SOST* gene, is secreted by osteocytes and suppresses bone formation. On the other hand, GLP1 has been found to reduce *SOST* (sclerostin) mRNA levels, which is known to inhibit bone formation through suppression of the Wnt/nt Wnt known to inhibit bone formation through suppression of tin mRNA and *COL1* [15]. On the other side of events, GLP1 has been known to regulate osteoclast functions. In particular, it has been found that GLP1 agonists are able to reduce serum levels of C-terminal cross-linked telopeptide of type I collagen (CTX-1) and urinary deoxypyridinoline (DPD)/creatinine ratio [16], thus inhibiting osteoclast action. Further on, GLP1 is able to decrease bone resorption through the calcitonin-dependent pathway [17]. The aforementioned mechanisms are summarized in Figure 2.

**Figure 3:**
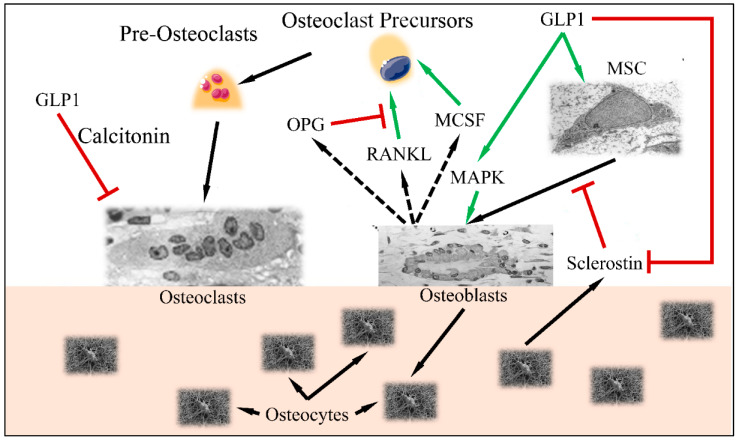
The balance between osteoblast and osteoclast functions: GLP1 induces MSC differentiation to osteoblasts, through the MAPK pathway. Osteocytes, produce sclerostin, which is inhibited by GLP1. Normally, stimulated osteoblasts produce MCSF, RANKL and OPG, which stimulate osteoclast precursors in order to retain bone formation balance (i.e., osteoblast and osteoclast functions remain equilibrated). GLP1 induces MAPK and osteoblast function as well as inhibits osteoclast function through the calcitonin-dependent pathway (Legend: GLP1: Glucagon-like peptide 1, MSC: Mesenchymal Stem Cells, MAPK mitogen-activated protein kinase, MCSF: *macrophage colony stimulating factor*, RANKL: Receptor activator of nuclear factor kappa-Β ligand, OPG: Osteoprotegerin. Green arrows imply stimulation; red arrows imply inhibition; black arrows imply cellular stimulation) (Inspired and reproduced from Schiellerup et al. (2019) [21] under the Creative Common License CC BY 3.0, link: https://www.frontiersin.org/articles/10.3389/fendo.2019.00075/full, accessed on 28 January 2022. MSC is reproduced under CC BY 3.0 License from https://en.wikipedia.org/wiki/Mesenchymal_stem_cell, Osteoblast is reproduced under the CC BY 3.0 License from https://en.wikipedia.org/wiki/Osteoblast, Osteoclast is reproduced under the CC BY-SA 3.0 License from https://en.wikipedia.org/wiki/Osteoclast, Osteocytes are reproduced from https://en.wikipedia.org/wiki/Osteocyte. All images last accessed on 24 January 2022).

It is apparent that GLP1 functions between a delicate balance between bone formation and bone resorption (Figure 3). Recent studies have indicated that GLP1R agonists are able to regulate the differentiation of mesenchymal stem cells to osteoblasts (reducing the differentiation of those cells to adipocytes) [18]. This has been found to take place through activation of the MAPK and PKC pathways [19], as well as the PKA/PI3K/AKT/GSK3T pathways [20].

### 1.2. Diabetes and Bone Metabolism

It has been confirmed that antidiabetic drugs, especially thiazolidinediones (TZDs) and insulin, reduce bone density and increase the risk of fracture [22,23]. TZD, also known as pioglitazone, is a synthetic peroxisome proliferator-activated receptor γ (PPARγ) stimulator, and it is a potent insulin sensitizer with multiple mechanisms of action. These drugs increase glucose consumption in muscle tissues, reduce the levels of glycerol, free fatty acids and triglycerides and increase insulin sensitivity in the muscle, adipose tissue and liver. Moreover, pioglitazone inhibits hepatic gluconeogenesis, resulting in decreased endogenous glucose production [24]. Τhe mechanisms by which basal insulin contributes to the regulation of hyperglycemia is through the reduction of hepatic glucose production and the suppression of lipolysis. According to a previous study, diabetic patients, mainly women who were treated with (TZDs) for an extended period, had an increased risk of fracture [25], which points out that deregulated insulin levels probably are related to increases in the occurrence of fractures [26].

On the other hand, incretin hormones have the ability to increase insulin secretion in response to nutrient ingestion [27]. Glucagon-like peptide-1 receptor agonists (GLP1RAs) stimulate insulin secretion from β cells, reduce β cell apoptosis, suppress glucagon secretion from α cells, increase liver insulin sensitivity, slow down gastric emptying and induce satiety [28]. Recently, GLP1RAs, including exenatide, liraglutide, lixisenatide, albiglutide, dulaglutide and semaglutide, are widely used for the treatment of type 2 diabetes mellitus (T2D). These factors can be classified as short-acting (e.g., exenatide and lixisenatide, twice daily) and long-acting (e.g., albiglutide, dulaglutide, exenatide and liraglutide, once weekly), which continuously activate the GLP1 receptor (GLP1R) at recommended doses [29]. Increasing evidence has shown that GLP1RAs are beneficial for bone metabolism [30,31], while no large-scale, long-term trials are available. So far, there is only little evidence on the effects of GLP1RAs on bone density in humans. Meanwhile, in addition to decreasing blood glucose in diabetic patients, these drugs can cause relevant weight loss, which may result in a reduction of bone density. Furthermore, several studies found that these agents can promote bone formation and inhibit bone resorption, while the underlying molecular mechanisms have not been elucidated [32].

In the present study, we reviewed the literature in order to investigate the relation between GLP1 and bone metabolism. Meanwhile, we attempted to review the clinical relevance of GLP1RAs in bone metabolism.

## 2. Materials and Methods

**Figure 4:**
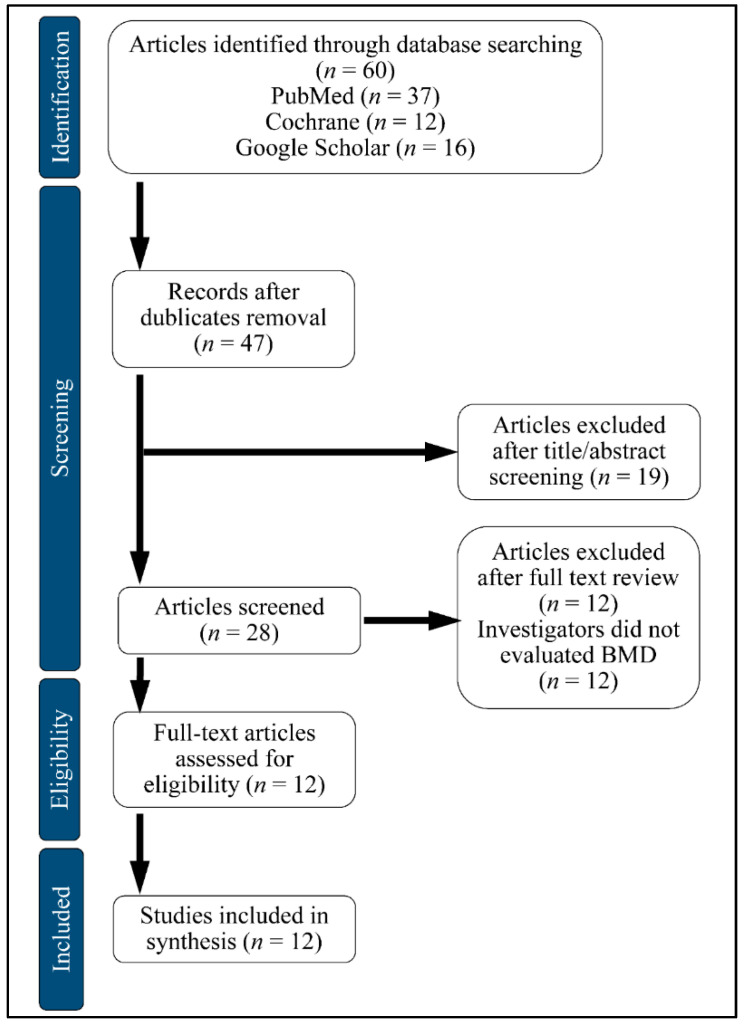
Flow chart of study selection.BMD: Bone Mineral Density.

We searched PubMed, Google Scholar and Cochrane Central Register of Controlled Trials (CENTRAL). A computerized search for the databases was accomplished by using Medical Subject Heading and entry terms such as: Glucagon-like Peptide-1 Receptor Agonists (GLP1RAs), Bone Mineral Density (BMD), Diabetes Mellitus (DM). We have included publications in English and excluded review papers and studies in the form of comments or author articles, from October 2019 to January 2020. We also included animal studies. The studies included in this systematic review were performed in Denmark, the Netherlands, China and the United States. The flowchart of the selection is presented in Figure 4.

## 3. Results

There is an intense debate on whether GLP1RAs can minimize the risk of fracture [33]. Yet, they seem to be an ideal treatment option for diabetics with high-risk fractures due to the low risk of hypoglycemia [34,35]. The main findings of relevant studies are summarized in Table 1 and Table 2.

### 3.1. Human Studies

For the studies included in this systematic review, the sample size ranged from 61 to 69, the mean age of type 2 diabetes mellitus (T2D) was 54 years and the study duration varied from 24 weeks to 2 years.

The study population was consistent, but heterogeneity in doses and duration of therapy was observed. Specifically, in Bunck et al. (2011) [36], metformin-treated patients were included, who were randomized to receive exenatide twice daily; in Li et al. (2015) [37], patients newly diagnosed with T2D assigned to exenatide treatment at a dose of 5 μg twice daily were enrolled; in Gilbert et al. (2016) [38], patients receiving liraglutide treatment 1.8 mg/day and liraglutide 1.2 mg/day were examined; while in Iepsen et al. (2015) [39], 37 healthy obese women who were treated with liraglutide 1.2 mg/day were included. All the four randomized controlled trials (RCTs) consisted of an intervention group and a control group. Generally, the effect of liraglutide and exenatide was investigated in the clinical trials (Table 1).

According to the study by Bunck et al. (2011), BMD was not affected by exenatide, significant weight loss was observed in the group of T2D, while alkaline phosphatase (ALP), calcium (Ca^+2^) and phosphate (P^+4^), as the markers of bone metabolism and calcium homeostasis, remained unaffected after a 44-week treatment [36]. Similarly, in a two-center, randomized, parallel-group clinical trial, the effects of exenatide on BMD and bone turnover markers including osteocalcin (OC), C-telopeptide of type I collagen (CTX-1) and tartrate-resistant alkaline phosphatase 5b (TRAcP5b) were compared with insulin or pioglitazone treatment (*n* = 62, newly diagnosed T2D patients). In the study, no improvement in BMD or bone turnover markers was detected, despite the amelioration of glucose levels, after 24 weeks of treatment [37] (Table 1).

In Gilbert et al. (2016), a multicenter trial examined the effectiveness of liraglutide (1.2 and 1.8 mg/day) versus glimepiride monotherapy in T2D. After 52 and 104 weeks of treatment, there was no apparent difference in mean total BMD in the diabetic patients [38]. However, Iepsen et al. (2015) demonstrated that liraglutide increased bone formation marker *N*-terminal propeptide of type1procollagen (P1NP) by 16% in a group of healthy obese women, while the bone resorption marker CTX-1 was not affected [39] (Table 1).

With respect to fracture risk, Mabilleau et al. (2014) revealed, in a meta-analysis of 28 RCTs, that GLP1RA had no impact on the risk of bone fracture in T2D, as compared to the patients treated with other antidiabetic drugs [40]. This was followed by a meta-analysis conducted by Su et al. (2015), where 16 RCTs were included in order to investigate the risk of bone fractures associated with liraglutide or exenatide, compared to placebo or other active drugs. Data revealed that liraglutide was associated with a decreased risk of fracture, while exenatide treatment was remarkably associated with an increased risk of fracture [41]. On the other hand, Zhang et al. (2018) conducted a network meta-analysis based on 54 RCTs where 28,353 out of 49,602 participants were treated with exenatide, liraglutide, semaglutide, dulaglutide, albiglutide or lixisenatide, while the others were treated with other antidiabetic drugs or placebo. It was found that exenatide was remarkably associated with a lower risk of fracture, as compared to placebo. Exenatide is the best option concerning the risk of fracture, followed by dulaglutide, liraglutide, albiglutide, lixisenatide and semaglutide [42]. No association was found between GLP1RAs and fracture risks in both studies conducted by Driessen et al. (2015) [43,44] (Table 1).

The difference in doses and duration of therapy with GLP1RAs and the difference in the number of trials included in meta-analyses may have led to the above-mentioned conflicting findings [27]. Further studies are needed to elucidate the effect of different GLP1RAs on bone formation [47].

### 3.2. Animal Studies

In opposition to what has been recognized in humans, animal models of T2D are associated with increased bone formation and decreased bone resorption. In ovariectomy-induced (OVX) osteoporosis, old rats receiving exendin-4 at a dose of 3 or 10 μg/kg/day for 16 weeks were associated with increased serum ALP levels, which is a significant biomarker of bone formation, and inhibited bone resorption. Such changes might be induced by increasing OPG/RANKL ratio. On the contrary, serum CTX-1, a major biomarker for the estimation of bone resorption rate, was decreased by exendin-4 [16] (Table 2).

In the study conducted by Nuche-Berenguer et al. (2009), several bone characteristics in T2D and insulin resistant (IR) rat models were evaluated. They investigated the effect of GLP1 treatment on T2D and IR rats, and found that following GLP1 treatment, the OPG/RANKL ratio was increased in T2D and IR rats as compared to normal rats. These findings indicated that GLP1 could be a salutary therapeutic factor for increasing bone formation [8]. It has also been mentioned that exendin-4 administration in T2D and IR rats promoted bone formation via the Wnt signaling pathway [11]. In another study by Nuche-Berenguer et al. (2011) based on a hyperlipidic model, exedin-4 also induced a significant increase in the tibias’ OPG/RANKL ratio and osteocalcin, as compared to the control [45] (Table 2).

GLP1 receptor is expressed in thyroid C cells and induces calcitonin secretion. GLP1R^−/−^ mice had an increased level of urinary deoxypyridinoline (DPD), which was associated with increased bone resorption. When treating these GLP1R^−/−^ mice with calcitonin, moderation of the increased urinary DPD was observed. As a consequence, it can be deduced that GLP1 inhibits bone resorption in a calcitonin dependent way [17]. Additionally, it has been noticed that exendin-4 decreased the mRNA and protein levels of SOST-sclerostin in osteocyte-like MLO-Y4 cells, and also reduced the serum sclerostin level in T2D OLEFT rats. On the other hand, the serum levels of osteocalcin and femoral BMD were increased by exendin-4. Exendin-4 might raise BMD by reducing the expression of SOST/sclerostin in osteocytes in T2D [46]. Meanwhile, GLP1 may also guide cell differentiation via regulating the ΜAΡΚ and Wnt signaling pathways to promote RunX2 activity. Moreover, GLP-1RAs have been demonstrated to possess anabolic effects on bones in ovariectomized animal models without diabetes, whereas exenatide is associated with bone mass elevation in both trabecular and cortical bones [20].

## 4. Discussion

The present systematic review investigated the effect of GLP1RAs on BMD as well as attempted to find the clinical relevance of GLP1RAs treatment in bone metabolism. Previous studies have shown that patients with diabetes mellitus are associated with an increased risk of bone fractures [48]. It is well-known that diabetes causes chronic complications such as neuropathy and retinopathy, while bone fractures are recognized as a significant diabetes complication with high mortality and morbidity [49]. The connection between diabetes and the frequency of fractures as well as the underlying mechanisms have not been clarified. It has been confirmed that anti-hyperglycemic remedies, especially TZDs and insulin, known for maintaining glycemic levels, can affect bone metabolism by reducing bone density and increasing the risk of fracture [48]. GLP1RAs are widely used for the treatment of T2D and seem to be an ideal treatment therapy option for diabetes patients with high-risk fractures due to the low risk of hypoglycemia [50]. The pleiotropic effect of GLP1RAs has facilitated clinicians investigating their relationship to bone metabolism.

Although, some human studies have reported that GLP1RAs have no impact on BMD or the turnover markers, all agree on the fact that administration of GLP1A can be beneficial for bone metabolism. On the other hand, several clinical trials showed that GLP1RAs and other antidiabetic drugs were found to be associated with either increased or decreased risks of fracture. A very recent report, indicated that the use of exenatide and dulaglutide in T2D patients, did not have any effect on BMD after 52 weeks of treatment, while the same cohort manifested a significant improvement of BMD when treated with insulin glargine [51]. In general, all available studies agree on one point; that the use of GLP1 agonists in the treatment of diabetes is beneficial for both the disease in questions as well as bone metabolism [52]. Similarly, a recent report suggested that GLP1 agonists when administered to T2D patients with concurrent cardiovascular disease, lead to significant reduction of hospitalizations, as well as kept bone metabolism unaffected [53]. In addition, a recent interesting review on the subject indicated that the administration of GLP1 agonists had an effect on the suppression of chronic inflammation by reducing the levels of inflammatory cytokines [54]. The same work indicated that GLP1 agonists lead to a significant increase in femoral and vertebral bone mass, confirming previous findings on the role of GLP1 agonists.

The aforementioned clinical studies and meta-analyses had some limitations. First of all, the number of control groups in many studies was too small to draw firm conclusions. Therefore, the effects of GLP1RAs on BMD and on fracture risk would have been adequately demonstrated if the size of the study population was larger. Second, the follow-up duration in many trials was insufficient to strongly support the association between GLP1RA treatments and bone fractures. It is also noteworthy that the age of the populations under investigation was younger than the typical age of the osteoporosis population. Furthermore, there was no distinction between men and women in the samples’ cohorts. Therefore, the protective effect of GLP1RAs on bone fracture risk was potentially undermined by these factors (i.e., age and gender). In addition, bone fracture was not the primary endpoint in the included meta-analyses, leading to insufficient registration and recording.

On the other hand, most animal studies have shown an increase in BMD and bone turnover markers, which was in contrast to that observed in human studies. In rodent models, administration of GLP1 or its analogue exendin-4 for 3 days increased BMD and augmented the expression of osteoblast markers through diverse mechanisms, such as interacting with the Wnt pathway or increasing the OPG/RANKL ratio in normal, diabetic and hyperlipidic rats [8,11,45]. In addition, it has been indicated that GLP1R may inhibit bone formation through a calcitonin dependent pathway in thyroid C cells by stimulating calcitonin releasing [17]. Another mechanism through which exendin-4 increases BMD is reducing the expression of SOST/sclerostin in MLO-Y4 cells, which leads to increased serum levels of osteocalcin, decreased serum levels of sclerostin and increased femoral BMD in T2D rats [46].

In fact, differences in the expression levels of GLP1 might be attributable to the inconsistent results between animal studies and clinical trials [16]. Another explanation for the discrepancy between human and animal studies might reside in the use of much higher drug doses in the experimental models, which provided a more favorable effect than those administered in the human population. In addition, the duration of treatment in clinical trials was not sufficiently long to examine the anabolic, anti-resorptive and protective effects of GLP1RAs on bone and fracture risk. Furthermore, not all human studies contained information about bone status or measured the serum markers of bone formation and resorption at baseline, and thus the possible action of GLP1RAs was not highlighted [30].

The limitations arising from the present systematic review included the lack of sample’s homogeneity (both human and animal populations), a small number of registered participants and short duration of follow-up treatment. Another limitation of the present review is the methodological heterogeneity as different GLP1RAs were examined, which have different pharmacokinetic profiles.

## 5. Conclusions

Patients suffering from DM are at high risk of bone fracture. This systematic review concluded that antidiabetic medication could intervene with skeletal physiology. GLP1RAs belong to the incretin family of antidiabetic drugs. In addition to their known glucose-independent lowering action, it has been reported that these agents are associated with beneficial skeletal effects. Overall clinical data indicated that GLP1RAs have a neutral impact on bone health and do not affect the rates of fracture. However, further double-blind RCTs are required to draw more meaningful and significant conclusions on their efficacy on BMD.

## Figures and Tables

**Table 1:** (HUMANS) Published human and animal research on the impact of GLP1 on bone metabolism.

Paper/Reference	Study	Subjects	Study Duration	Measurements	Fracture Risk	BMD	Bone Metabolism/Turnover Markers	Main Results
Bunck et al. (2011) [36]	RCT	69 metformin-treated T2D patients with exenatide vs. insulin glargine	44 weeks	BMD, ALP, Ca, P		↔	ALP: ↔ Ca: ↔ P: ↔	BMD, serum markers of bone metabolism and calcium homeostasis remained unaffected by exenatide treatment
Li et al. (2015) [37]	RCT	62 newly diagnosed and drug-naïve patients with T2D, treated with exenatide vs. insulin vs. pioglitazone	24 weeks	HbA1C, BMD, CTX, OC, TRAcP5b		↔	HbA1C: ↑ CTX: ↔ OC: ↔ TRAcP5b: ↔	Exenatide had no impact on bone turnover markers or BMD
Gilbert et al. (2016) [38]	RCT	61 T2D patients, treated with liraglutide and glimepiride	52 or 104 weeks	BMD		↔		Liraglutide did not affect total BMD
Iepsen et al. (2015) [39]	RCT	37 healthy obese women aged 46 ± 2 years treated with or without liraglutide	52 weeks	CTX-1, P1NP			CTX-1: ↔ P1NP: ↑	Liraglutide increased P1NP by 16% but did not change CTX-1
Mabilleau et al. (2014) [40]	Meta-analysis	A meta-analysis, 28 RCTs were identified T2D, treated with either a GLP1Ra or another antidiabetic drug	24 weeks (at least)	Incidence of bone fracture	↔			GLP1RA was not associated with reduced fracture risk
Su et al. (2015) [41]	Meta-analysis	A meta-analysis, 16 RCTs were identified, Liraglutide or exenatide treatnebt vs. placebo or other diabetic drugs	N/A	Risk of bone fracture	Liraglutide: ↓ Exenatide: ↑			Liraglutide might reduce the risk of bone fractures while exenatide might increase the risk of bone fractures
Zhang et al. (2018) [42]	Meta-analysis	Network meta-analysis, 54 RCTs were identified, GLP1Ra vs. other hypoglycemic drugs vs. placebo	N/A	Fracture risk	Exenatide: ↓			Exenatide was associated with a decreased risk of bone fracture
Driessen et al. (2015) [43]	Case Control	A case-control study of NIAD users vs. GLP1RA users	N/A	Risk of fractures	↔			GLP1RA was not associated with fracture risk
Driessen et al. (2015) [44]	Case Control	Population-based cohort, T2D patients with at least one prescription for NIAD GLP1RA vs. non-GLP1RA	N/A	Bone fracture risk	↔			GLP1RA was not associated with decreased bone fracture

Abbreviations: **↑** increase; **↓** decrease; **↔** neutral; BMD, bone mineral density; ALP, Alkaline Phosphatase; CTX, C-terminal Telopeptide; OC: Osteocalcin; TRAcP5b, Tartrate-resistant acid phosphatase 5b; CTX-1, carboxy-terminal cross-linked telopeptide of type *1* collagen; P1NP, procollagen-1 N-terminal peptide; GLP1RA, glucagon-like peptide-1 receptor agonists; T2D, type 2 diabetes mellitus; RCTs, randomized control trials; NIAD, non-insulin antidiabetic drug; HbA1C, glycosylated hemoglobin; N/A, Not Available.

**Table 2:** (ANIMALS) Published human and animal research on the impact of GLP1 on bone metabolism.

Paper/Reference	Subjects	Measurements	BMD	Bone Metabolism/Turnover Markers	Main Results
Ma et al. (2013) [16]	Old ovariectomy rats	DPD/creatinine, CTX-1, ALP, OC, P1NP, Coll-1, Runx2, OPG/RANKL		DPD/creatinine: ↓ CTX-1: ↓ ALP: ↑, OC: ↑, P1NP: ↑, Coll-1: ↑ Runx2: ↑ OPG/RANKL: ↑	Exendin-4 prevented osteopenia by increasing bone formation markers and decreasing bone resorption markers
Nuche-Berenguer (2009) [8]	T2D rats, insulin-resistant rats vs. normal rats	BMD OC, OPG/RANKL	↑	OC: ↑, OPG/RANKL: ↑ (T2D+IR)	GLP1 increased bone formation
Nuche-Berenguer (2010) [11]	T2D rats, insulin-resistant rats vs. normal rats	BMD, OPG/RANKL, TRAP5b, OC	↑	OPG/RANKL: ↑ (T2D+IR) TRAP5b: ↓ OC: ↑	Exendin-4 exerted osteogenic effects
Nuche-Berenguer (2011) [45]	Hyperlipidic and hypercaloric rats	OPG/RANKL OC		RANKL/OPG: ↑ OC: ↑	GLP1 and Exendin-4 reversed bone alterations in hyperlipidic rats
Yamada et al. (2017) [17]	GLP1R knockout mice vs. wild-type mice	Calcitonin, DPD		DPD: ↓	GLP1 inhibited bone resorption in a calcitonin dependent way
Kim et al. (2013) [46]	4-week-old male T2D OLEFT rats with saline vs. OLEFT rats with exendin-4 vs. LETO control rats with saline	mRNA expression SOST/sclerostin Sclerostin OC TRAP5D BMD	↑	mRNA expression: ↓ SOST/sclerostin: ↓ Sclerostin: ↓ OC: ↑ TRAP5D: ↔	Exendin-4 might raise BMD by reducing the expression of SOST/sclerostin in osteocytes
Sun et al. (2015) [20]	5-month-old female nondiabetic and OVX wistar rats, Sham+vehicle vs. OVX+vehicle vs. OVX+exendin-4	BMD Runx2 ALP Coll-1 PPARγ C/EΒΡα P38 P42/44 Β-catenin proteins	↑	Runx2: ↑ ALP: ↑ Coll-1: ↑ PPARγ: ↓ C/EΒΡα: ↓ P38: ↑ P42/44: ↑ Β-catenin proteins: ↑	Exendin-4 exerted bone-preserving effects in OVX rats

Abbreviations: ↑ increase; ↓ decrease; ↔ neutral; BMD, bone mineral density; T2D, type 2 diabetes mellitus; CTX-1, C-telopeptide of type I collagen; ALP, alkaline phosphatase; OC, osteocalcin; OPG/RANKL, osteoprotegerin/receptor activator of nuclear factor—κΒ ligand; GLP1R, glucagon-like peptide-1 receptor; DPD, deoxypyridinoline; OLETF rats, Otsuka Long–Evans Tokushima Fatty rats; LETO rats, Long–Evans Tokushima Otsuka (LETO) rat; OVX, ovariectomized; TRAcP5b, tartrate-resistant alkaline phosphatase 5b; Runx2, runt-related transcription factor 2; Coll-1, collagen 1; PTH, parathyroid hormone; PPARγ, peroxisome proliferator—activated receptor γ.

## Data Availability

Not applicable.
